# In This Issue

**DOI:** 10.1002/cfg.296

**Published:** 2003-06


**Comparative and Functional Genomics**
*Comp Funct Genom* 2003; **4**: 285.
Published online in Wiley InterScience (www.interscience.wiley.com). **DOI:** 10.1002/cfg.296

### In this issue

### Performance assessment of kernel density clustering for expression data

Guoping Shu and colleagues have devised a novel kernel density approach for clustering of expression data that they have tested against other approaches such as hierarchical and K-means clustering.

### Reproducibility assessment of independent component analysis for microarray data

David Kreil and David MacKay present a detailed study of the robustness of independent component analysis of expression ratio data from microarrays.

### Gene recognition in the *Saccharomyces cerevisiae* genome

In a mathematical approach to annotating the *Saccharomyces cerevisiae* genome, Liaofu Luo and colleagues have defined a new metric to aid in identifying coding ORFs. They have also analysed the distribution of ORFs and ORF lengths in the yeast genome.

### Methylation analysis of several tumour suppressor genes in uveal melanomas

Michael Zeschnigk *et al.* have found methylated alleles of two tumour suppressor genes on chromosome 3 in a subset of uveal melanomas.

### Mapping functional associations in the *Drosophila melanogaster* genome

Ioannis Iliopoulos *et al.* have used gene fusion analysis, followed by filtering to select for non-paralogous pairs, to identify pairs of genes in the *Drosophila* genome that have a functional relationship.

### Simple sequence repeats database of the human genome (SSRD)

SSRD is a collation of data on single sequence repeats in the human genome. Subbaya Subramaniam and co-workers present this data as a series of interactive tables.

### Association of interleukin gene mutations with asthma

Maxim Freidin and colleagues report on their studies of an *IL4* mutation and an *IL5* mutation in a population of atopic bronchial asthma patients, from Tomsk, Russia.

### Featured organism: honey bee genomics

Jay Evans and Daniel Weaver present an overview of the current status of bee genomics, and their hopes for the future, in the light of the impending bee genome sequencing project.

### Conference calendar

We present a round-up of genomics related conferences planned for August–November 2003.

Copyright © 2003 John Wiley & Sons, Ltd.

